# Fatty acid transporters in the porcine conceptus and early placenta and the effects of polyunsaturated fatty acids on trophoblast cells

**DOI:** 10.1038/s41598-026-51024-w

**Published:** 2026-05-02

**Authors:** Agnieszka Blitek, Magdalena Szymanska

**Affiliations:** InLife Institute of Animal Reproduction and Food Research, Polish Academy of Sciences, Trylinskiego 18, 10-683 Olsztyn, Poland

**Keywords:** Pig, Pregnancy, Placenta development, Fatty acid transporters, Polyunsaturated fatty acids, Trophoblast cells, Cell biology, Developmental biology, Diseases, Molecular biology, Physiology

## Abstract

This study aimed to determine whether fatty acids (FAs) may affect the function of the early porcine placenta. First, the expression of FA transporters (CD36, SLC27A) in conceptuses and placentae of days 10–11, 12–13, 15–16, 18–20, 25, and 30 pregnant gilts (n = 5–8 per group) was examined using Real-time PCR, Western blot, and immunohistochemistry. Then, primary trophoblast (pTr) cells from days 15–16 conceptuses were exposed to n-6 and n-3 polyunsaturated FAs (PUFAs) to study prostaglandin (PG) synthesis and the expression of genes related to FA action, angiogenesis, steroidogenesis, and lipid transport. Furthermore, pTr cell proliferation and adhesion in response to PUFAs were determined colorimetrically. Increased mRNA expression of *CD36*, *SLC27A1*, and *SLC27A2* was detected in days 18–25 placentae compared with days 10–13 conceptuses. SLC27A4 and *SLC27A6* expression was greater in days 10–11 spherical than in days 15–16 elongated conceptuses. SLC27A1, SLC27A4, and SLC27A6 were localized at the placenta-endometrium interface. PUFAs of n-6 series elevated PGE2 and PGI2 synthesis, whereas n-3 PUFAs stimulated PGE2 but inhibited PGI2 output. All PUFAs up-regulated the mRNA expression of *CPT1A*, a rate-limiting enzyme of FA β-oxidation. Moreover, docosahexaenoic acid (DHA) increased *FABP5*, *SLC27A4*, *LDLR* (lipoprotein receptor), and proangiogenic *ANGPT1* and *ANGPTL4* mRNA expression. DHA and arachidonic acid stimulated pTr cell proliferation, while linoleic and eicosapentaenoic acids increased cell adhesion. These results are the first demonstrating dynamic changes of FA transporter expression in peri-implantation conceptuses and developing placentae of the pig and indicate FA uptake by the early placenta. Furthermore, PUFAs may support placenta development by modulating gene expression, increasing PGE2 level, and promoting trophoblast cell viability and adhesion.

**Supplementary Information:**

The online version contains supplementary material available at 10.1038/s41598-026-51024-w.

## Introduction

Placenta constitutes the primary maternal–fetal interface and carries out an array of diverse functions for the maintenance and support of normal pregnancy. Placental transport efficiency is a key determinant of the fetal growth^1–3^. In the pig, placenta development intensifies at the period of conceptus (the embryo and associated extraembryonic membranes) elongation that occurs on days 10 to 12 of pregnancy when spherical (1–2 mm) conceptuses rapidly remodel into tubular (> 10 mm) morphology, and finally to long filamentous (> 100 mm) forms^4–6^. In this multiparous species, which possesses a true epitheliochorial placenta, elongation of the conceptus is the primary factor affecting the size of the placenta as it determines the amount of uterine space and maternal resources available for the fetus^3^. Initial adhesion of conceptus trophoblast to the uterine epithelium starts at approximately day 13 of gestation followed by a more stable attachment on days 15–16, with the formal implantation occurring on days 18 to 25^6,7^. Then, porcine placenta continues to grow rapidly until day 60^1,3^.

Developing conceptus requires substantial amounts of nutrients to support rapid cellular growth and activity^2,8^. Numerous proteins, amino acids, glucose, lipids, vitamins, and minerals are transported from the maternal endometrium^6,8^. Among lipids, fatty acids (FAs) are crucial for the cell membrane composition, eicosanoid synthesis, intracellular signaling and metabolic activity^9,10^. Moreover, various FAs may affect gene expression and protein activity to regulate cell growth and survival, angiogenesis and inflammatory response^10–12^. A group of FAs, named polyunsaturated fatty acids (PUFAs) have been described as important for embryonic/fetal growth and placenta development in humans^9,10,13^. PUFAs of n-3 and n-6 series are essential FAs; they must be delivered with food because mammals are not able to synthesize them de novo. Alpha-linolenic acid (ALA; C18:3n-3) and its long-chain derivatives docosahexaenoic acid (DHA; C22:6n-3) and eicosapentaenoic acid (EPA; C20:5n-3) belong to n-3 PUFAs. Linoleic acid (LA; C18:2n-6) and its most physiologically important long-chain derivative arachidonic acid (ARA; C20:4n-6) are classified as n-6 PUFAs^14^. Of interest, n-3 and n-6 PUFAs compete with each other for their metabolism and produce compounds with diverse physiological and pathological activities. In general, n-6 PUFA-derived eicosanoids express pro-inflammatory and pro-angiogenic effects, whereas n-3 PUFAs and their eicosanoid derivatives mostly promote anti-inflammatory and anti-angiogenic effects^10,14^.

The exact mechanism of how FAs cross the plasma membrane has not been fully elucidated. Both simple diffusion (mainly short- and medium-chain FAs) and facilitated transport via membrane proteins (mainly long-chain FAs) are possible. As for the latter, fatty acid translocase (FAT; also known as CD36) and fatty acid transport proteins (FATP; encoded by *SLC27A* genes) were indicated as mediators of FA transport in the placenta^15–17^. Out of six FATP family members, FATP1-4 and FATP6 were localized in the placenta of humans^16,18^ and mice^19^. Among livestock species, the expression of FA transporters was demonstrated for bovine^20^, ovine^21^, and porcine^22–24^ placentae. In the pig, the gene expression analysis of nutrient transporters in day 85 placenta revealed *SLC27A4* and *SLC27A6* mRNA expression in trophoblast cells of the folded placental-maternal bilayer, the structure that develops during pregnancy to facilitate the transport of nutrients^22^. Decreased *SLC27A2* and *SLC27A4* mRNA expression was detected in day 65 porcine placentae of cloned fetuses compared with fetuses developed following artificial insemination and was accompanied by reduced levels of FAs in the allantoic fluid^24^. Moreover, lower mRNA expression of *CD36*, *SLC27A1*, and *SLC27A4* was detected in full-term placentae of obese sows than their lean counterparts that resulted in compromised placental lipid transport, decreased placental efficiency, and reduced litter size and litter weight^23^. All these data indicate that placental FA transport in the second half of pregnancy in the pig is important for fetal development and litter quality. Much less data however, is available regarding FA transporters and the role of FAs during the period of early stages of embryo/fetus and placenta development in this species. Intriguingly, significant up-regulation of *SLC27A6* mRNA expression was observed on days 9–11 of pregnancy during the initial transformation of porcine conceptuses from spherical to tubular forms and was accompanied by a greater abundance of lipid metabolism-related transcripts^25^. Moreover, feeding gilts with different FA composition on days 1 to 60 of gestation influenced the growth and development of the offspring^26^. These limited data indicate a possible role of FAs for conceptus development and placenta formation in the pig. In particular, rapid alteration in conceptus morphology on days 10 to 12 of pregnancy occurs through cellular migration and remodeling of trophectoderm and endoderm including their elongation and cytoskeletal reorganization^5^. The subsequent conceptus implantation and initial placenta development on days 15 to 30 of gestation involve intense proliferation and migration of cells as well as changes in their morphology and secretory properties^5,6,22^. All these processes are energy-demanding and accompanied by modifications in prostaglandin (PG) synthesis and the mRNA/protein expression^6,27,28^. Therefore, the present study was undertaken (1) to examine the mRNA and protein expression of CD36 and SLC27A in the porcine conceptus and placenta on days 10 to 30 of pregnancy, (2) to localize FA transporters at the placenta-endometrium interface on day 30 of gestation, and (3) to determine whether PUFAs of n-3 and n-6 series may influence placenta function. To achieve the latter goal, porcine primary trophoblast (pTr) cells were exposed to selected PUFAs followed by analyses of PGE2 and PGI2 concentrations and the mRNA and/or protein expression of factors engaged in PG synthesis, steroidogenesis, angiogenesis, lipid transport as well as FA binding, action, and metabolism (full list of genes is listed in Supplementary Table S1). Porcine pTr cell proliferation and adhesion in response to PUFAs were also examined.

## Results

### The mRNA expression of FA transporters in conceptus and placenta of early pregnant gilts

**Fig. 1:**
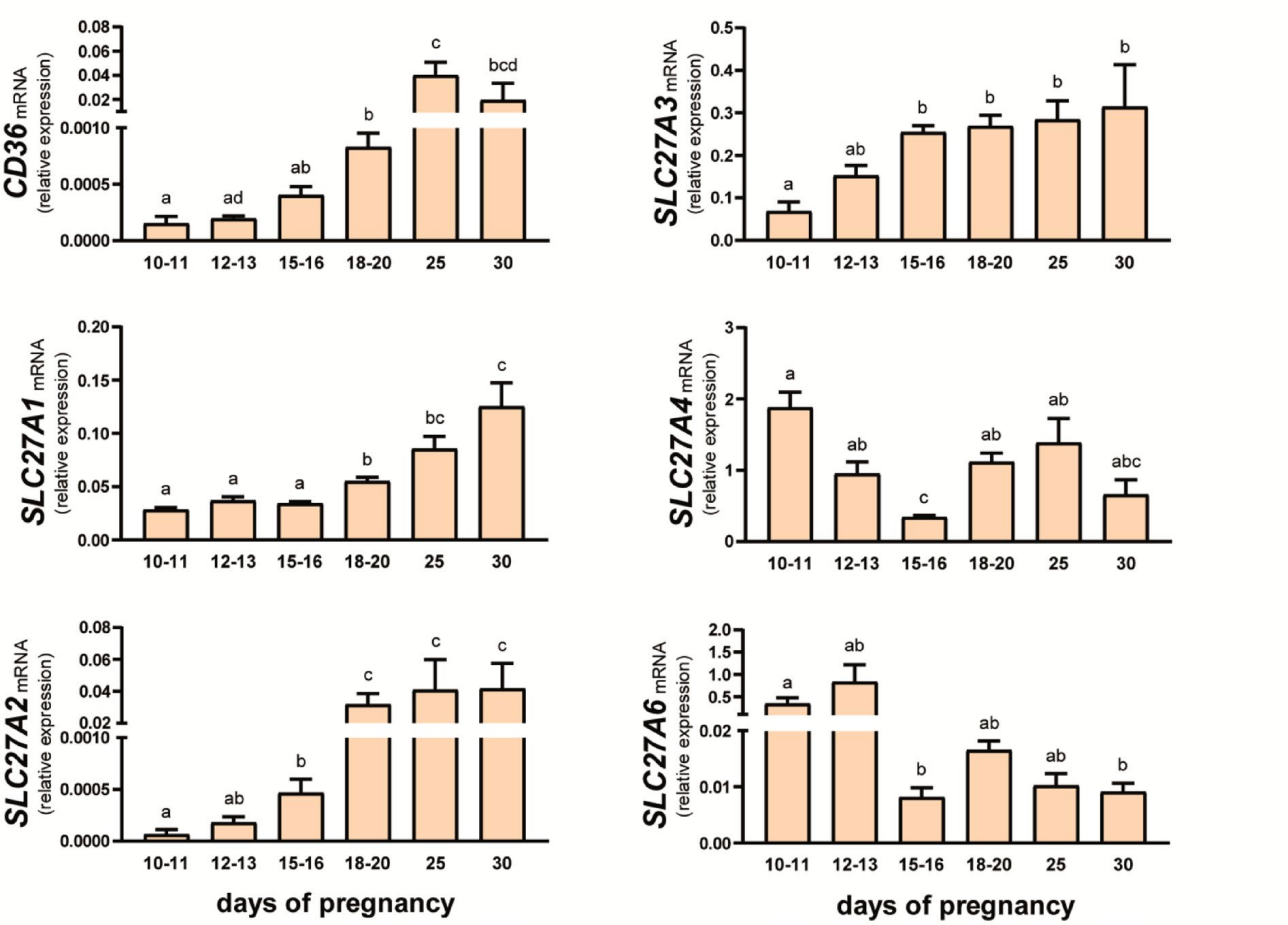
The mRNA expression of *CD36*, *SLC27A1*, *SLC27A2*, *SLC27A3*, *SLC27A4*, and *SLC27A6* in conceptuses and placentae collected on days 10–11, 12–13, 15–16, 18–20, 25, and 30 of pregnancy. Values from Real-time PCR were normalized to geometric averaging of glyceraldehyde-3-phosphate dehydrogenase (*GAPDH*) and hypoxanthine phosphoribosyltransferase 1 (*HPRT1*) mRNA expression. Data are presented as the mean ± SEM (n = 5–8). Bars marked with various letters are different (*p* < 0.05).

The mRNA expression of all FA transporters varied in conceptus and placenta during the examined period of early pregnancy (Fig. 1). *CD36*, *SLC27A1*, and *SLC27A2* showed similar profiles of mRNA expression with increased levels detected in the placenta on days 18–20, 25, and 30 compared with conceptuses from days 10–11 (*p* < 0.05 for *CD36* and *SLC27A1*, and *p* < 0.01 for *SLC27A2*). Moreover, *SLC27A1* mRNA expression was greater in the placenta on day 30 compared with days 18–20 (*p* < 0.001), whereas *SLC27A2* mRNA expression was greater in days 18 to 30 placentae compared with days 15–16 conceptuses (*p* < 0.001). *SLC27A3* mRNA expression was up-regulated in elongated conceptuses from days 15–16 and placenta tissue from days 18 to 30 of gestation compared with spherical conceptuses collected on days 10–11 (*p* < 0.01). By contrast, a decrease in *SLC27A4* mRNA expression was detected in days 15–16 elongated conceptuses compared with days 10–11 spherical conceptuses (*p* < 0.001) and with days 12–13 filamentous conceptuses (*p* < 0.05). After this drop, *SLC27A4* mRNA expression increased and was greater in placentae collected on days 18 to 25 of gestation compared with days 15–16 conceptuses (*p* < 0.01). In turn, *SLC27A6* mRNA expression decreased in days 15–16 conceptuses compared with those collected on days 10–11 of pregnancy (*p* < 0.05) and remained lower also in the placenta from day 30 (*p* < 0.05 compared with days 10–11 conceptuses).

### The protein abundance of FA transporters in conceptus and placenta of early pregnant gilts

**Fig. 2:**
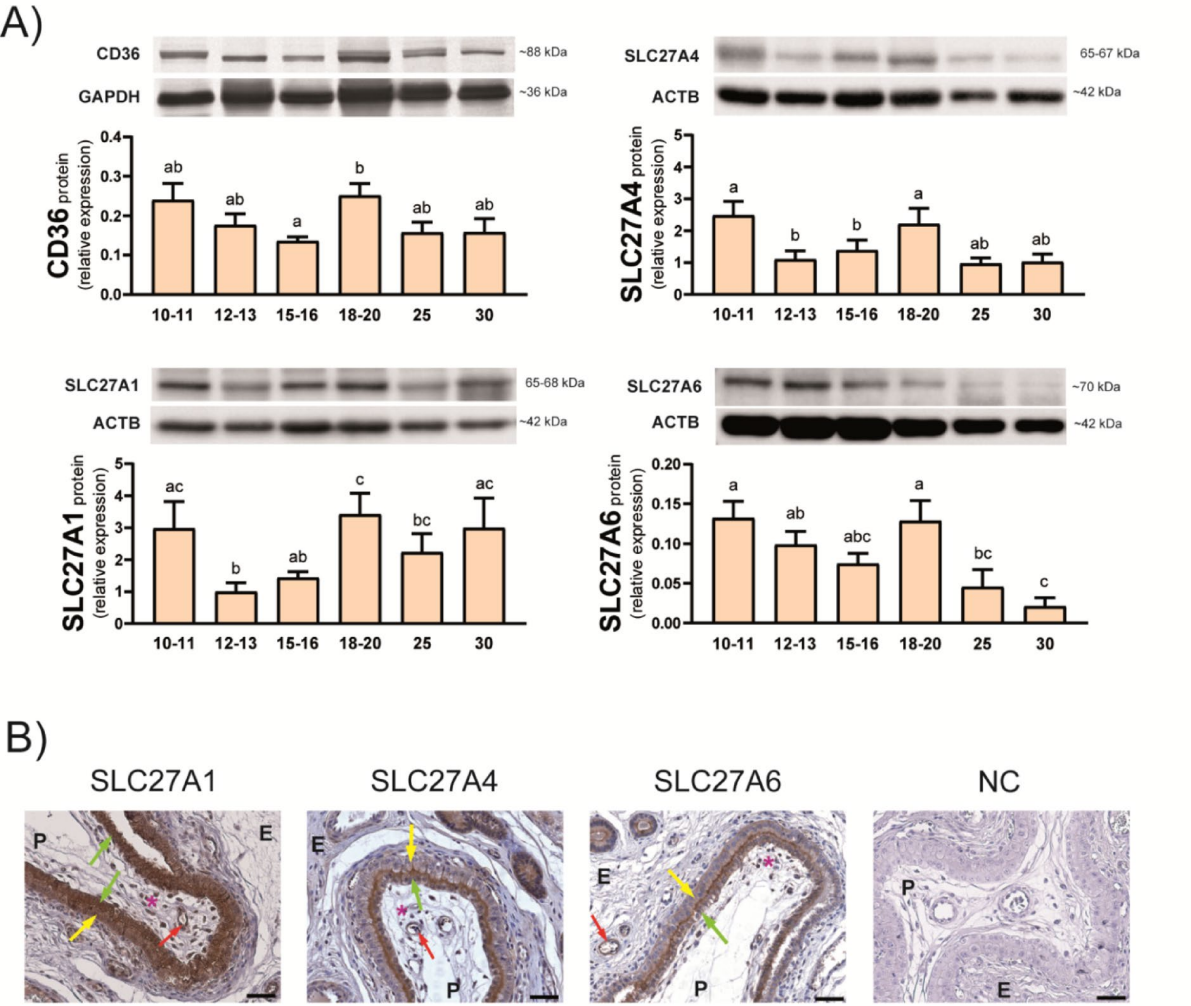
The protein expression of CD36, SLC27A1, SLC27A4, and SLC27A6 in conceptuses and placentae collected on days 10–11, 12–13, 15–16, 18–20, 25, and 30 of pregnancy (**A**) and the localization of SLC27A1, SLC27A4, and SLC27A6 proteins at the placenta-endometrium interface on day 30 of pregnancy (**B**). In (**A**) fragments of representative blots are presented; full blots are included in Supplementary Figs S1 and S2. Values from densitometric analyses of bands were normalized to β-actin (ACTB) or glyceraldehyde-3-phosphate dehydrogenase (GAPDH) and presented as the mean ± SEM (n = 5–6). Bars marked with various letters are different (*p* < 0.05). In (**B**) tissue sections were stained with hematoxylin (blue staining) to visualize nuclei. For the negative control (NC), primary antibodies were replaced with the rabbit IgG. Green arrows—placenta trophoblast; yellow arrows—luminal epithelium of the endometrium; red arrows—blood vessels; asterisk—connective tissue. P, placenta; E, endometrium. Scale bars: 50 μm.

Similar to endometrial tissue^29^, application of various anti-SLC27A2 and anti-SLC27A3 antibodies did not result in the detection of specific protein bands in conceptus and placenta samples using Western blot technique. Thus, the protein expression of CD36, SLC27A1, SLC27A4, and SLC27A6 was further determined. As demonstrated in Fig. 2A, a variable protein abundance of the examined transporters in conceptus and placenta was observed.

The abundance of CD36 protein was almost twofold greater in days 18–20 placenta tissue compared with days 15–16 conceptuses (*p* < 0.05). Both SLC27A1 and SLC27A4 proteins showed down-regulation of their expression in days 12–13 filamentous conceptuses compared with days 10–11 spherical conceptuses (*p* < 0.05 and *p* < 0.01, respectively). Then, the abundance of both proteins was greater in the placenta from days 18–20 compared with conceptuses collected on days 12–13 and 15–16 of pregnancy (*p* < 0.05). SLC27A6 protein abundance was lower in the placenta from day 30 of gestation compared with conceptuses from days 10–13 (*p* < 0.05) and with the placenta from days 18–20 (*p* < 0.5) of pregnancy.

### Localization of FA transporters at the placenta-endometrium interface

SLC27A1, SLC27A4, and SLC27A6 proteins were detected using immunohistochemistry (IHC) in the placenta and maternal endometrium of day 30 pregnant pigs (Fig. 2B). SLC27A1 protein was strongly expressed in both trophoblast cells of the placenta and luminal epithelial cells of the endometrium. In turn, more apparent immunostaining for SLC27A4 and SLC27A6 proteins was observed in placenta trophoblast than in the endometrial epithelial cells. Moreover, the connective tissue and blood vessels stained positively for SLC27A1, SLC27A4, and SLC27A6 proteins.

### Effect of n-6 and n-3 PUFAs on PGE2 and PGI2 secretion from pTr cells

**Fig. 3:**
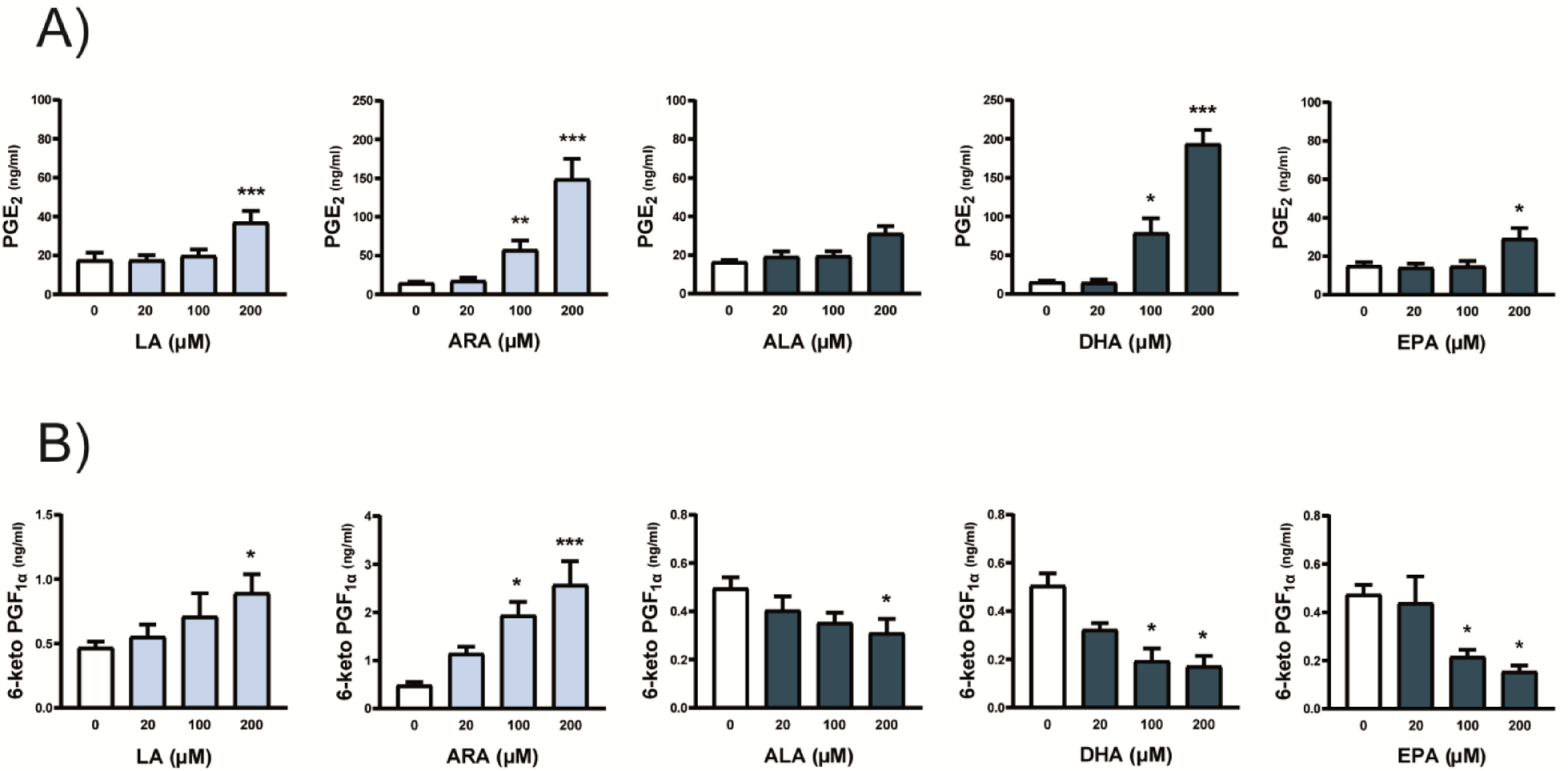
Effect of n-6 (linoleic acid [LA] and arachidonic acid [ARA]; light blue bars) and n-3 (α-linolenic acid [ALA], docosahexaenoic acid [DHA], and eicosapentaenoic acid [EPA]; dark blue bars) PUFAs on prostaglandin E2 (PGE2; **A**) and 6-keto prostaglandin F1α (6-keto PGF1α, a stable metabolite of prostaglandin I2 [PGI2]; **B**) concentrations in the culture medium. Porcine trophoblast cells were exposed to increasing doses (20, 100, and 200 μM) of each PUFA for 24 h. Data are presented as the mean ± SEM (n = 4–6). Asterisks indicate differences compared with non-treated cells (*, *p* < 0.05; **, *p* < 0.01; ***, *p* < 0.001).

Examined PUFAs significantly influenced PGE2 and PGI2 secretion from pTr cells (Fig. 3). PGE2 concentration in medium increased in response to the highest dose of LA (17.6 ± 3.8 vs. 37.0 ± 6.0 ng/ml; *p* < 0.001) and ARA (14.6 ± 1.5 vs. 148.5 ± 26.4 ng/ml; *p* < 0.001; Fig. 3A). The middle dose of ARA also stimulated PGE2 release (a fourfold increase; *p* < 0.01). Among n-3 PUFAs, DHA and EPA but not ALA affected PGE2 levels in the culture medium. The treatment of pTr cells with 100 and 200 μM DHA resulted in fivefold (*p* < 0.05) and 12.5-fold (*p* < 0.001) increases in PGE2 concentrations. Only the highest dose of EPA stimulated PGE2 secretion (15.1 ± 1.8 vs. 29.2 ± 5.4 ng/ml; *p* < 0.05).

Similar to PGE2, 6-keto PGF1α (a stable metabolite of PGI2) secretion from pTr cells was increased by n-6 PUFAs (Fig. 3B). Greater concentrations of 6-keto PGF1α in medium were observed in response to the highest dose of LA (0.47 ± 0.05 vs. 0.89 ± 0.14 ng/ml; *p* < 0.05) and two doses of ARA (fourfold increase for 100 μM, *p* < 0.05 and 5.4-fold increase for 200 μM, *p* < 0.001). In contrast to PGE2, n-3 PUFAs inhibited the secretion of 6-keto PGF1α into culture medium. Decreased concentrations of a PGI2 metabolite were detected in response to 100 and 200 μM DHA (2.6- and threefold decreases, respectively; *p* < 0.05) or EPA (2.2- and threefold decreases, respectively; *p* < 0.05). The addition of the highest dose of ALA also resulted in a lower concentration of 6-keto PGF1α compared with non-conditioned medium (0.31 ± 0.06 vs. 0.49 ± 0.05 ng/ml; *p* < 0.05).

### Effect of n-6 and n-3 PUFAs on the expression of factors involved in PG synthesis, FA action, and angiogenesis in pTr cells

**Fig. 4:**
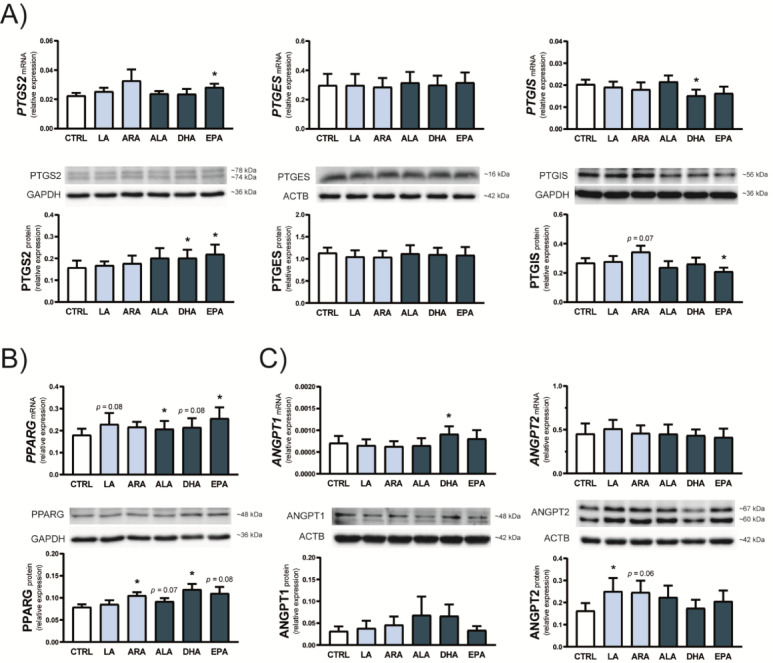
Effect of n-6 (linoleic acid [LA] and arachidonic acid [ARA]; light blue bars) and n-3 (α-linolenic acid [ALA], docosahexaenoic acid [DHA], and eicosapentaenoic acid [EPA]; dark blue bars) PUFAs on the relative mRNA and protein expression of prostaglandin synthesis enzymes (prostaglandin-endoperoxide synthase 2 [PTGS2], prostaglandin E synthase [PTGES], and prostaglandin I2 synthase [PTGIS]; **A**), peroxisome proliferator-activated receptor gamma (PPARG; **B**), and angiopoietin 1 and 2 (ANGPT1 and ANGPT2; **C**) in porcine trophoblast cells. Cells were exposed to 200 μM of PUFAs for 24 h. Values from Real-time PCR were normalized to geometric averaging of glyceraldehyde-3-phosphate dehydrogenase (*GAPDH*) and hypoxanthine phosphoribosyltransferase 1 (*HPRT1*) mRNA expression. Values from densitometric analyses of bands were normalized to β-actin (ACTB) or GAPDH. Two bands detected for ANGPT2 were sum up before normalization. Fragments of representative blots are presented; full blots are included in Supplementary Figs S3 and S4. All numerical data are presented as the mean ± SEM (n = 4–5). Asterisk indicates the difference compared with the control value (CTRL; *, *p* < 0.05).

The mRNA expression of *PTGS2* in pTr cells was stimulated by EPA (*p* < 0.05 compared with the control value), while the protein abundance increased in response to both DHA and EPA (*p* < 0.05; Fig. 4A). None of PUFAs affected the mRNA or protein expression of PTGES. The addition of DHA to the medium inhibited *PTGIS* mRNA expression in pTr cells (*p* < 0.05). PTGIS protein abundance decreased in the presence of EPA (*p* < 0.05) while tended to be greater after the addition of ARA (*p* = 0.07).

Greater expression of *PPARG* mRNA in pTr cells was observed in the presence of ALA and EPA (*p* < 0.05 compared with non-treated cells), while LA and DHA tended (*p* = 0.08) to increase *PPARG* mRNA expression (Fig. 4B). PPARG protein abundance was elevated in the presence of ARA and DHA (1.3- and 1.4-fold increases, respectively; *p* < 0.05). ALA and EPA tended (*p* = 0.07 and *p* = 0.08, respectively) to increase PPARG protein abundance.

The mRNA expression of *ANGPT1* was stimulated by DHA (*p* < 0.05; Fig. 4C), while ANGPT1 protein abundance was not affected. In contrast to ANGPT1, protein but not mRNA expression of ANGPT2 was affected by PUFAs, with LA showing a stimulatory effect (*p* < 0.05 compared with the control value) and ARA tended to up-regulate ANGPT2 protein abundance (*p* = 0.06).

### Effect of n-6 and n-3 PUFAs on the mRNA expression of selected genes in pTr cells

**Fig. 5:**
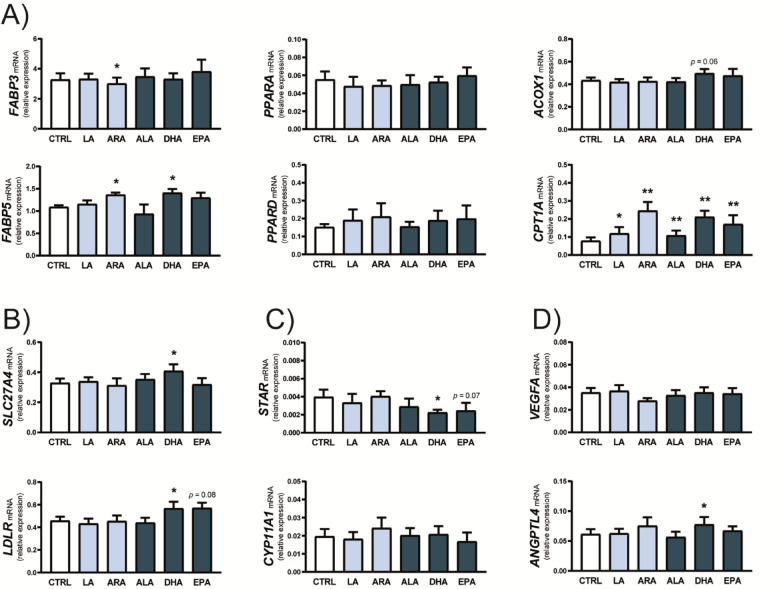
Effect of n-6 (linoleic acid [LA] and arachidonic acid [ARA]; light blue bars) and n-3 (α-linolenic acid [ALA], docosahexaenoic acid [DHA], and eicosapentaenoic acid [EPA]; dark blue bars) PUFAs on the relative mRNA expression of genes involved in fatty acid binding (*FABP3*, *FABP5*), action (*PPARA*, *PPARD*), and metabolism (*ACOX1*, *CPT1A*; **A**), transmembrane lipid transport (*SLC27A4*, *LDLR*; **B**), steroidogenesis (*STAR*, *CYP11A1*; **C**), and angiogenesis (*VEGFA*, *ANGPTL4*; **D**) in porcine trophoblast cells. Cells were exposed to 200 μM of PUFAs for 24 h. Values from Real-time PCR were normalized to geometric averaging of glyceraldehyde-3-phosphate dehydrogenase (*GAPDH*) and hypoxanthine phosphoribosyltransferase 1 (*HPRT1*) mRNA expression and presented as the mean ± SEM (n = 3–5). Asterisks indicate differences compared with the control value (CTRL; *, *p* < 0.05; **, *p* < 0.01).

Examined PUFAs differentially affected the mRNA expression of genes related to FA binding, action, and metabolism in pTr cells (Fig. 5A). The presence of ARA decreased *FABP3* mRNA expression (*p* < 0.05 compared with the control value), while ARA and DHA stimulated *FABP5* mRNA expression by 25% and 30%, respectively (*p* < 0.05). Neither *PPARA* nor *PPARD* mRNA expression changed after the addition of PUFAs. The *ACOX1* mRNA expression tended to increase in the presence of DHA (*p* = 0.06). Moreover, all examined PUFAs markedly up-regulated *CPT1A* mRNA expression in pTr cells (*p* < 0.05).

Expression of genes related to transmembrane lipid transport, steroidogenesis, and angiogenesis was differently affected by PUFAs. Both *SLC27A4* and *LDLR* mRNA expression increased after the addition of DHA (*p* < 0.05; Fig. 5B), whereas EPA tended to stimulate *LDLR* mRNA expression (*p* = 0.08). The mRNA expression of *STAR*, in turn, was decreased in the presence of DHA (*p* < 0.05 compared with the control cells) and tended to be lower after the exposure of cells to EPA (*p* = 0.07; Fig. 5C). Greater expression of *ANGPTL4* mRNA was observed in pTr cells cultured with DHA (*p* < 0.05; Fig. 5D). None of the examined PUFAs affected *CYP11A1* and *VEGFA* mRNA expression.

### Effect of n-6 and n-3 PUFAs on pTr cell proliferation and adhesion

**Fig. 6:**
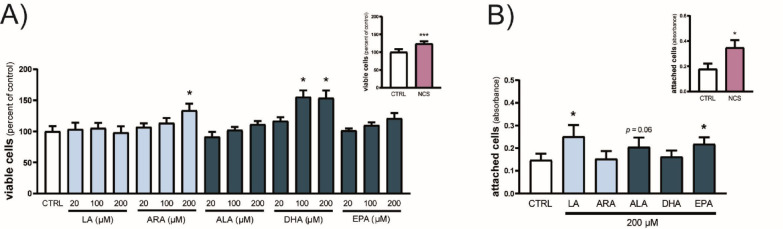
Effect of n-6 (linoleic acid [LA] and arachidonic acid [ARA]; light blue bars) and n-3 (α-linolenic acid [ALA], docosahexaenoic acid [DHA], and eicosapentaenoic acid [EPA]; dark blue bars) PUFAs on the proliferation (**A**) and adhesion (**B**) of porcine trophoblast cells. Newborn calf serum (NCS; 10%; pink bar in insets) was used as a positive control. For proliferation analysis, cells were exposed to increasing doses (20, 100, and 200 μM) of PUFAs for 24 h followed by the addition of CellTiter 96 AQueous One Solution Reagent. For adhesion analysis, cells were pre-treated with PUFAs or NCS for 60 min, followed by an 3.5 h long incubation in Millicoat Cell Adhesion Strips with final staining procedure using crystal violet. Data are presented as the mean ± SEM (n = 4–7). Asterisks indicate differences compared with the control value (CTRL; *, *p* < 0.05; ***, *p* < 0.001).

The number of viable pTr cells was greater after 24 h of culture with ARA at the dose of 200 μM (an increase of 34%; *p* < 0.05) and with DHA at doses of 100 and 200 μM (increases of 55% and 54%, respectively; *p* < 0.05) compared with control cells (Fig. 6A). The incubation with LA and EPA resulted in a greater number of cells attached to fibronectin-coated strips compared with control cells (increases by 70% and 50%, respectively; *p* < 0.05; Fig. 6B). Moreover, ALA tended to increase the adhesion of cells (*p* = 0.06). Newborn calf serum (NCS) used as a positive control in both assays significantly stimulated cell proliferation (an increase of 23.7%; *p* < 0.001) and adhesion (an almost twofold greater number of cells; *p* < 0.05) compared with not-exposed cells.

## Discussion

The majority of embryonic mortality in the pig occurs between days 10 to 30 of pregnancy^30^; this is at the time when the maternal recognition of pregnancy, conceptus implantation, and the initial formation of the placenta occur^1,5,27^. Rapid remodeling of the conceptus on days 10 to 12^5^, the formation of a stable adhesion of conceptuses to the maternal endometrium on days 15 to 20^4,6,7^, and further intense growth and folding of the placenta after day 20^22,31^ of pregnancy require membrane motility and energy supply. Alterations in the expression of various lipid-related genes have been previously demonstrated for elongating porcine conceptuses^25,32,33^ and early placentae^34^. Moreover, global characterization of proteins in the uterine lumen of early pregnant gilts demonstrated that among proteins with increased concentrations on day 13 compared with day 10 was salivary lipocalin^35^. This protein binds many lipids, including FAs, as reported for the equine uterus and conceptus^36^. Additionally, FAs were among the top three classes of metabolites identified in the porcine uterine fluid on days 10 to 16 of pregnancy^37^. These findings indicate the role of FAs during this pivotal period of early pregnancy.

As we demonstrated in the present study, the mRNA expression of *SLC27A4* and *SLC27A6* was greater in spherical than elongated conceptuses indicating that these transporters are important for conceptus transformation before implantation. Increased mRNA expression of *CD36*, *SLC27A1*, *SLC27A2*, and *SLC27A3* was detected in placentae collected on days 18 to 30 of gestation, the period when the formal implantation was accomplished, compared with conceptuses from the pre-implantation period of pregnancy (days 10 to 13). Thus, transporters encoded by *CD36*, *SLC27A1*, *SLC27A2*, and *SLC27A3* seem to participate in FA uptake required for the growth of the placenta during and after conceptus implantation. In support of this, *CD36*, *FATP1*, *FATP2*, and *FATP3* were expressed in days 40, 65, 95, and full-term porcine placentae^38^. Genes encoding FA transporters were among differentially expressed genes identified in peri-implantation bovine^39^ and ovine^40^ conceptuses; *SLC27A1*, *SLC27A2*, or *SLC27A6* were up-regulated in elongated compared with spherical conceptuses. In contrast to early embryonic development, no data is available describing FA transporter expression in early placenta of ruminants; however, the abundance of *SLC27A1*, *SLC27A2*, *SLC27A3*, and *SLC27A4* transcripts increased in ovine placentomes between days 70 and 135 of pregnancy^21^.

To our knowledge, this is the first study demonstrating both mRNA and protein expression profiles of FA transporters in peri-implantation conceptus and the early placenta of livestock species. As we showed here, SCL27A1, SLC27A4, and SLC27A6 proteins were clearly visible in trophoblast cells at the placenta-endometrium interface as well as in the underlying connective tissue and blood vessels. It indicates active FA transport in the early pig placenta. The present results are consistent with the localization of SLC27A4 and CD36 proteins in the cytotrophoblast layer of the porcine full-term placenta^38^. Our further Western blot analysis showed that protein expression of FA transporters varied along with porcine conceptus or placenta development; however, the changes were not as dynamic as those observed for mRNA expression. In general, CD36, SLC27A1, and SLC27A4 protein abundance showed a transient decrease in conceptuses on days 12–13 or 15–16 followed by a greater expression in the placenta on days 18–20 of gestation. It points to the role of these transporters in the uptake of FAs during the period of conceptus implantation. In turn, protein expression of SLC27A6 was lower in placenta tissue from days 25 and 30 than in early conceptuses and was in accordance with changes in *SLC27A6* mRNA expression. Such a profile points to a role of SLC27A6 during the pre-implantation conceptus development rather than during the placenta growth.

While the profile of SLC27A4 protein expression coincided with that of mRNA, both CD36 and SLC27A1 transporters showed non-overlapping profiles of mRNA and protein expression. Such a difference may result from various processes influencing the mRNA stability or function, including mRNA modifications and mRNA-protein interactions. At the protein level, activation of protein degradation pathways or post-translational modifications are also possible^41^. Furthermore, microRNAs, which function as post-transcriptional regulators of gene expression, may be responsible for the non-overlapping profiles of FA transporter mRNA and protein expression; several microRNAs were identified in the porcine conceptuses and placentae on days 10 to 20 of pregnancy^42^. Nevertheless, current results clearly confirm the presence of a membrane protein system that may facilitate FA uptake by the placenta during the first month of pregnancy in the pig. However, further research is needed to determine mechanisms regulating FA transporter expression and activity in placenta cells.

PUFAs are of critical importance for fetal growth; their rate of utilization increases with fetus age, reaching the maximal demand right before term^43^. Importantly, the placenta requires a constant and abundant source of energy to supply the needs for its own growth and function at all gestational stages^16,44^. Various FAs may affect the viability, gene expression, and the secretory activity of trophoblast cells^10,13,45–47^. Supplementation of pregnant mice with n-3 or n-6 PUFAs improved both embryonic and placental growth^48^. Enriching the sows’ diet with n-3 PUFAs during the first two months of pregnancy increased pre-weaning growth and weaning weight of piglets^26^. Notably, LA, ARA, ALA, DHA, and EPA were detected in day 30 porcine embryos and supplementation of pregnant sows with dietary DHA resulted in elevated levels of this FA in embryonic tissue^49^. It points to the transfer of PUFAs in the early pig placenta. However, there is no data describing how PUFAs affect placenta cells during this period of gestation. Therefore, we used pTr cells to examine the impact of n-3 and n-6 PUFAs on PG synthesis, gene expression, cell proliferation and adhesion; processes that are crucial for the pregnancy establishment^5,6,27,28^.

Porcine conceptuses synthesize and release substantial amounts of PGs^5,28^. PGE2 is essential for the maternal recognition of pregnancy and influences endometrial preparation for conceptus implantation^28^, while PGI2 stimulates pTr cell proliferation and adhesion^50^ and modulates endometrial angiogenesis^51^. As we demonstrated here, the treatment of pTr cells with LA or ARA resulted in elevated concentrations of PGE2 and PGI2 in culture medium. In particular, ARA showed a dose-dependent action on the output of both PGs, with significant increases observed at higher concentrations. Such an effect is consistent with the well described role of ARA as the main precursor for PG synthesis^14^ and with our previous results showing greater PGE2 and PGI2 release from the porcine endometrium in the presence of this FA^29^. Furthermore, application of feeding diets containing a high proportion of n-6 PUFAs increased blood plasma levels of PGE2 in early pregnant gilts^52^. Of interest, n-3 PUFAs differentially affected PGE2 and PGI2 release from pTr cells with their dose-dependent stimulatory action on PGE2 concentrations and inhibitory effect on PGI2 content in the culture medium (the present data). PGI2 is well described as a positive modulator of angiogenesis and vascular function^51,53^; thus, the stimulatory effect of LA and ARA and the inhibitory action of LA, DHA, and EPA on PGI2 synthesis observed here are consistent with pro-angiogenic and anti-angiogenic properties of n-6 and n-3 PUFAs, respectively. In support of this, ARA stimulated while EPA reduced 6-keto PGF1α release from human endothelial cells^54^. In contrast to our present data, n-3 PUFAs decreased PGE2 synthesis in the bovine endometrium and trophoblast tissue^55^. Therefore, the effect of PUFAs on PG secretion may be cell/tissue- and species-specific.

In the current study, substantial changes in PGE2 or PGI2 levels in the medium in response to PUFAs were not accompanied by similar changes in the expression of enzymes involved in PG synthesis. Therefore, differences in PG concentrations in pTr cells may result from changes in the activity rather than in the expression of PTGES and PTGIS, the terminal enzymes in PGE2 and PGI2 synthesis pathways, respectively. Similar results were previously described for the porcine endometrium exposed to PUFAs^29^ or to cytokines^56^. Greater concentrations of PGE2 in response to PUFAs accompanied by decreased mRNA expression of *PTGES* was detected in bovine endometrial cells^57^. Nevertheless, the present results indicate that n-6 and n-3 PUFAs may support pregnancy progression by increasing PGE2 levels, which is crucial for both endometrial remodeling and corpus luteum maintenance^27,28^. Moreover, greater levels of PGI2 in response to n-6 PUFA may be important for the development and function of the vascular bed in the placenta.

After entering the cell, FAs are bound by FABPs and trafficked to various compartments for FA metabolism, β-oxidation, signal transduction, ligand activation, or transfer to the fetus^45,58^. We previously demonstrated FABPs expression in trophoblast cells of day 15 conceptuses^59^ and day 25 placentae^34^. The present study revealed that *FABP5* mRNA expression in pTr cells was stimulated by ARA and DHA, while *FABP3* mRNA expression decreased in the presence of ARA. Similarly, greater *FABP5* but not *FABP3* mRNA expression was detected in human term placenta trophoblast cells in response to a mixture of LA and oleic acid^60^. Moreover, the mRNA expression of *FABP5* but not *FABP3* increased in the porcine placenta between days 40 and 95 of pregnancy^38^. Thus, FABP5 seems to be the main binding protein responsible for the intracellular distribution of PUFAs entering trophoblast cells.

PPARs function as transcription factors and key regulators of lipid metabolism and energy homeostasis, and various FAs are natural ligands of PPARs^61^. All three PPAR isoforms are present in porcine conceptuses and placentae, and their activation influences trophoblast cell proliferation, PGE2 synthesis, and the expression of genes involved in steroidogenesis and nutrient transport^34,59^. Furthermore, PPARG expression increases markedly in porcine conceptuses during their rapid elongation on days 10 to 12 of pregnancy^25,33^. We demonstrated here that among the studied PPARs, *PPARG* expression was increased by PUFAs. Therefore, PPARG may be considered as the main transcription factor participating in the action of PUFAs on gene expression in the porcine placenta.

Furthermore, we reported here that all examined PUFAs stimulated *CPT1A* mRNA expression in pTr cells. CPT1A protein is essential for FA transport into mitochondria for β-oxidation^62^. Therefore, PUFAs may participate in lipid-dependent energy homeostasis processes which are required for the cellular growth and remodeling of the porcine placenta. Of note, DHA may also increase the amount of available lipids, including FAs, by up-regulating the expression of their transporters as we demonstrated in the present study. Similarly, DHA was previously shown to stimulate *SLC27A4* expression in human trophoblast cells^63^.

Placental angiogenesis is critical for the feto-maternal exchange. PUFAs of both n-3 and n-6 series influenced angiogenesis with variable responses observed. In the human placenta, PUFAs stimulated *VEGF* and *ANGPTL4* mRNA expression^45,46^. In the present study, n-3 DHA increased pro-angiogenic *ANGPTL4* and *ANGPT1* mRNA expression while n-6 LA and ARA stimulated the anti-angiogenic ANGPT2 protein abundance. Thus, the ratio of n-3 and n-6 PUFAs reaching the placenta cells may be important for the course of angiogenesis-related processes. Further studies however, are required to evaluate how PUFAs affect the formation and/or stability of blood vessels in the porcine placenta.

The proper adhesion of trophoblast cells to the uterine epithelium and the subsequent cell proliferation are important for the placenta development^22,31^. In the present study, ARA and DHA stimulated pTr cell proliferation. Our results do not support the previous observation showing no effects of DHA on the proliferation of porcine pTr2 cell line^64^. However, the stimulatory effect of DHA on cell proliferation was demonstrated in bovine^65^ and rat^66^ ovarian cells. Moreover, the present report is the first showing the stimulatory effect of PUFAs on the adhesion of trophoblast cells. Although the exact mechanism has not been determined here, the impact of PUFAs on cell membrane fluidity may be responsible for the adhesive properties of the trophoblast cells. Overall, PUFAs appear to have a supportive role in the formation of the pig placenta by facilitating both proliferation and adhesion of trophoblast cells.

The main limitation of this research however, is the inability to describe the biological consequences of the changes observed in trophoblast cells for the placenta development and/or function. Moreover, some mechanisms controlling the abundance of FAs being available and taken up by the placenta may exist. Therefore, further studies using an animal model should be undertaken to examine processes and pathways regulated by PUFAs with their potential implications for pregnancy and fetal outcomes.

## Conclusions

The present study demonstrated the presence of FA transporters at the placenta-endometrium interface and reported variable profiles of their expression in the conceptus/placenta during early pregnancy in the pig. Furthermore, we showed that maternal n-3 and n-6 PUFAs may influence trophoblast cell function; in particular, PUFAs modulated the synthesis of PGE2 and PGI2 and the expression of factors involved in angiogenesis, lipid transport, and FA binding, action, and metabolism. Moreover, PUFAs promoted pTr cell proliferation and adhesion. All these results indicate that PUFAs may be actively transported by the pig placenta not only to support fetus growth and development but also to promote its own formation and function.

## Material and methods

### Animals and sample collection

Crossbred gilts (Polish Landrace x Duroc) used in this study were born and housed in a private farm under standard conditions defined by the breeder, in accordance with regulatory requirements. Animals were subjected to commercial breeding procedure, and the samples were collected post-mortem during the regular slaughter process. According to the Directive 2010/63/EU of the European Parliament and the Council of 22 September 2010 on the protection of animals used for scientific purposes as well as the Act of 15 January 2015 on the protection of animals used for scientific and educational purposes, the ethical review and approval are not required for this study. All experiments were performed in compliance with the ARRIVE guidelines.

For ex vivo examinations of FA transporter expression, 40 gilts of similar age, weight and genetic background from one commercial herd were used. After exhibiting two consecutive estrous cycles, gilts were bred 12 and 24 h after the estrus detection. The day of the second breeding was described as the first day of pregnancy. Gilts were slaughtered on days 10–11 (n = 8), 12–13 (n = 8), 15–16 (n = 8), 18–20 (n = 6), 25 (n = 5), and 30 (n = 5) of gestation. The number of animals was estimated based on both ethical guidelines for the use of animals for scientific purposes and similar experiments previously applied^29,34^, ensuring sufficient statistical power while reducing the number of animals. Conceptuses and placentae were collected based on the procedure previously described^42^. On days 10 to 16 of pregnancy, conceptuses were recovered by gently flushing each uterine horn with 20 ml of sterile phosphate-buffered saline (PBS; 137 mM NaCl, 27 mM KCl, 10 mM Na2HPO4, and 2 mM KH2PO4; pH 7.4) and assigned to the following groups/days: days 10–11 (spherical conceptuses), days 12–13 (filamentous conceptuses), and days 15–16 (elongated conceptuses). Conceptuses flushed from both uterine horns of each gilt on days 10 to 16 were pooled and treated as one sample. On days 18 to 30 of pregnancy, uterine horn of each gilt was cut open and the placenta was separated from one randomly selected embryo localized in the middle portion of the uterine horn. Conceptuses (days 10 to 16) and placentae (days 18 to 30) were snap frozen in liquid nitrogen and stored at − 80 °C for mRNA and protein analyses. For the IHC procedure, fragments of the uterus with attached placenta from day 30 pregnant gilts were fixed in 4% paraformaldehyde solution and embedded in paraffin.

For in vitro experiments, conceptuses were collected from 14 gilts (8 described above and 6 additional) slaughtered on days 15–16 of pregnancy and used for enzymatic isolation of pTr cells.

### Trophoblast cell isolation

Porcine pTr cells were isolated according to the procedure described earlier^67^. Briefly, trophoblast tissue was cut into small pieces and digested with 0.25% trypsin solution (Biomed, Lublin, Poland) for 30 min at 38 °C with gentle shaking. The cell suspension was filtered through two layers of gauze to remove undigested tissue fragments. After centrifugation (800x*g*, 10 min), cells were washed and suspended in Dulbecco’s Modified Eagle’s Medium (DMEM; D6046; Sigma-Aldrich, St. Louis, MO, USA) supplemented with 15 mM HEPES (H4034; Sigma-Aldrich) and antibiotics (100 IU/ml penicillin and 100 μg/ml streptomycin; Sigma-Aldrich), and counted. Depending on the experiment, pTr cells were cultured in 6-, 12-, or 96-well plates (Experiments 1 to 3) or subjected to adhesion assay (Experiment 4). All cell cultures were conducted at 37 °C in a humidified atmosphere of 5% CO2 and 95% air. The purity of pTr cell culture was assessed to be 90–95% based on microscopic observations.

### Experiment 1: effect of n-6 and n-3 PUFAs on PGE2 and PGI2 secretion by pTr cells

To verify whether porcine placenta may respond to PUFAs, pTr cells were suspended in the culture medium (DMEM supplemented with 15 mM HEPES, antibiotics, and 10% NCS [N4637; Sigma-Aldrich]) and seeded at a density of 2 × 10^5^ cells per well in 12-well culture plates (150628; Nunc™ Cell-Culture Treated Multidishes; Thermo Fisher Scientific; Waltham, MA, USA). After reaching 80–90% confluency, cells were gently washed and serum starved for 16 h. Then, the medium was discarded and cells were cultured in the basal medium (DMEM supplemented with 15 mM HEPES, antibiotics, and 1% FA-free bovine serum albumin [BSA; 03117057001; Roche Diagnostic GmbH, Mannheim, Germany]) only (control) or in the basal medium containing increasing doses (20, 100, and 200 μM) of the following PUFAs (all from Cayman Chemical): LA (90150), ARA (90010), ALA (90210), DHA (90310), or EPA (21908) for 24 h. PUFAs were prepared as described previously^47^. Doses of PUFAs were selected based on previously published data^64,65^. All treatments were performed in duplicate using cells isolated from six gilts. After incubation, media were collected and stored at − 40 °C until PGE2 and PGI2 concentration analyses.

### Experiment 2: effect of n-6 and n-3 PUFAs on the mRNA and/or protein expression of selected genes in pTr cells

To examine whether PUFAs may influence porcine placenta development and function, pTr cells were suspended in the culture medium and seeded at a density of 6 × 10^5^ per well in 6-well culture plates (140675; Thermo Fisher Scientific). After reaching 80–90% confluency, cells were serum starved for 16 h and exposed to the basal medium only (the same as in Experiment 1; control) or the basal medium containing LA, ARA, ALA, DHA, or EPA (200 μM each) for 24 h. The dose of PUFAs was chosen based on the results of Experiment 1. All treatments were performed in duplicate for both mRNA and protein expression using cells isolated from five gilts. After washing with PBS, pTr cells were treated with a TRI Reagent (TR118; Molecular Research Center, Cincinnati, OH, USA) for total RNA isolation or with the cell extraction buffer (10 mM Tris–HCl, pH 7.4; 100 mM NaCl, 1 mM EDTA, 1% Triton X-100, 0.1% sodium dodecyl sulfate, 0.5% sodium deoxycholate containing protease inhibitors [P-8340; Sigma-Aldrich]) for protein analyses. Samples were stored at − 80 °C for further processing.

### Experiment 3: effect of n-6 and n-3 PUFAs on pTr cell proliferation

To examine the effect of PUFAs on pTr cell proliferation, cells were suspended in the culture medium and seeded at a density of 5 × 10^3^ cells per well in 96-well culture plates (165306; Thermo Fisher Scientific). After reaching 60–70% confluency, cells were serum starved for 16 h. Then, cells were treated with the basal medium only (control) or the basal medium containing increasing doses (20, 100 and 200 μM) of LA, ARA, ALA, DHA or EPA for 24 h. Based on our previous results^34^, 10% NCS was used as a positive control for cell proliferation. The treatment was performed in triplicate using cells isolated from five gilts. After incubation, 20 μl of CellTiter 96 Aqueous One Solution Reagent (G3580; Promega, Madison, WI, USA) was added into each well for 2 h and the absorbance was measured at 490 nm wavelength using Epoch™ Microplate Spectrophotometer (BioTek Instruments, Inc., Winooski, VT, USA).

### Experiment 4: effect of n-6 and n-3 PUFAs on pTr cell adhesion

The effect of PUFAs on the adhesion of pTr cells was examined using Millicoat Cell Adhesion Strips coated with human fibronectin (ECM101; Millipore, Billerica, MA, USA). To this end, freshly isolated cells were suspended at a concentration of 3 × 10^5^ cells/ml in the basal medium only (control) or the basal medium containing LA, ARA, ALA, DHA, or EPA (200 μM each). After 60 min of pre-incubation, 100 μl of cell suspension containing medium only or medium with examined PUFAs was transferred into wells of Millicoat Cell Adhesion Strip (3 × 10^4^ cells/well) and incubated for 3.5 h at 37 °C in a humidified atmosphere of 5% CO_2_ and 95% air. Based on our previous results^67^, 10% NCS was used as a positive control for pTr cell adhesion. All treatments were performed in duplicate using cells isolated from seven gilts. After incubation, strips were gently washed with PBS containing Ca^2+^/Mg^2+^ and stained with 0.2% crystal violet as described earlier^67^. The absorbance was measured at 550 nm wavelength using Epoch™ Microplate Spectrophotometer.

### Enzyme immunoassay

To determine concentrations of PGE2 in incubation media (Experiment 1), a direct enzyme immunoassay method^68^ was used. Anti-PGE2 antibody (P5174; Sigma-Aldrich) developed in rabbits was applied at the dilution of 1:200. The sensitivity of the assay was 0.19 ng/ml, and the intra- and inter-assay coefficients of variation were 13.5% and 7.8%, respectively. To examine concentrations of 6-keto PGF1α (a stable metabolite of PGI2) in incubation media, a 6-keto PGF1α ELISA kit (515211; Cayman Chemicals) was used according to the manufacturer’s instructions. The sensitivity of the assay was 1.6 pg/ml, and the intra- and inter-assay coefficients of variation were 11.7% and 8.3%, respectively.

### Total RNA isolation and real-time PCR

Total RNA from conceptuses and placentae (ex vivo analysis) and from pTr cells (Experiment 2) was extracted using a Total RNA Prep Plus kit (031-100; A&A Biotechnology, Gdansk, Poland) and RNeasy Mini Kit (74104; Qiagen, Valencia, CA, USA), respectively, according to the manufacturers’ protocols. Samples were treated with DNase I (AMPD1; Sigma-Aldrich) and reverse transcribed using a High Capacity Reverse Transcription Kit (4374966; Applied Biosystems by Thermo Fisher Scientific), as described earlier^56^.

Diluted cDNA from RT-PCR was used to evaluate relative mRNA abundance of selected genes with an ABI Viia7 Sequence Detection System (Life Technologies Inc., Carlsbad, CA, USA). In order to examine the mRNA expression of *ACOX1*, *ANGPT1, ANGPT2, ANGPTL4*, *CD36, CPT1A*, *CYP11A1*, *FABP3, FABP5, LDLR, PPARA, PPARD, PPARG, PTGES, PTGIS, PTGS2, SLC27A1, SLC27A2, SLC27A3, SLC27A4, SLC27A6, STAR, VEGFA, ACTG1*, *HPRT1*, and *GAPDH*, 15 ng of cDNA was amplified using TaqMan Gene Expression Assays (Applied Biosystems by Thermo Fisher Scientific). All abbreviations of the examined genes, their full names, and the ID numbers of TaqMan probes are specified in Supplementary Table S1. Each PCR reaction was conducted in duplicates in 384-well plates under the following conditions: initial denaturation for 10 min at 95 °C, followed by 40 cycles of 15 s denaturation at 95 °C and then, 60 s of annealing at 60 °C. The control reactions in the absence of reverse transcriptase were carried out to check for genomic DNA contamination. Additionally, no template controls with nuclease-free water were performed to test for possible reagent contamination. Data from Real-time PCR were analyzed using the PCR Miner algorithm^69^. The NormFinder software version 0953^70^ was applied to select the most stable reference genes among *GAPDH*, *HPRT1*, and *ACTG1*. All expression data for each target gene were normalized against geometric averaging of *GAPDH* and *HPRT1*.

### Western blot

Conceptuses and placentae were homogenized using an ice-cold homogenization buffer (50 mM Tris–HCl, pH 8.0; 150 mM NaCl, 1% Triton X-100, 1 mM EDTA supplemented with protease inhibitor cocktail [Sigma-Aldrich]) in Lysing Matrix D (MP Biomedicals, Solon, OH, USA) with a FastPrep-24 instrument (MP Biomedicals). Homogenates were centrifuged for 10 min at 800x*g* at 4 °C. Cultured pTr cells were treated with the cell extraction buffer as described above (Experiment 2), detached from wells by scraping and transferred into microcentrifuge tubes. Then, the mixture was incubated on ice for 30 min with occasional vortexing and centrifuged at 13,000x*g* at 4 °C for 5 min. Supernatants from both tissue and cell extraction protocols were collected and stored for Western blot analyses.

Total protein extracts of the conceptus and placenta tissues (7.5 µg for SLC27A1; 10 µg for SLC27A4 and SLC27A6; 12 µg for CD36) or pTr cells (12 or 15 µg) were dissolved in SDS gel-loading buffer (50 mM Tris–HCl, pH 6.8; 4% SDS, 20% glycerol, and 2% β-mercaptoethanol). After heating at 95 °C for 5 min, samples were separated on 10% (ANGPT1, ANGPT2, CD36, PPARG, PTGIS, PTGS2, SCL27A1, SLC27A4, SLC27A6) or 12% (PTGES) SDS-PAGE. Separated proteins were electroblotted onto 0.45 µm pore size polyvinylidene difluoride membrane (IPVH00010; Merck Millipore Ltd Tullagreen, Carrigtwohill, Ireland) in transfer buffer (20 mM Tris–HCl, pH 8.2; 100 mM glycine, 20% methanol). TBS-T buffer (Tris-buffered saline, pH 7.4; 0.1% Tween-20) containing 5% nonfat dry milk was used for 1.5 h at room temperature to block nonspecific binding sites. Afterwards, membranes were incubated overnight with an appropriate primary antibody (Supplementary Table S2), washed with TBS-T, and incubated for 1 h with an anti-rabbit IgG-alkaline phosphatase antibody (A8024; Sigma-Aldrich; 1:20,000 dilution) or the Immun-Star™ goat anti-rabbit (GAR)-HRP conjugate (170-5046; Bio-Rad Laboratories, Inc., Hercules, CA, USA; 1:20,000) depending on the visualization procedure. For CD36 detection, immune complexes were visualized using a standard alkaline phosphatase visualization procedure. For ANGPT1, ANGPT2, PPARG, PTGES, PTGIS, PTGS2, SLC27A1, SLC27A4, and SLC27A6 detection, immune complexes were visualized using a Clarity Western ECL Substrate kit (Bio-Red Laboratories). Images were captured with the ChemiDoc™ Touch Imaging System and quantified using Image Lab™ Software Standard Edition, v. 6.0.0 Build 25 (both from Bio-Rad Laboratories). An internal control for protein loading was performed by re-blocking membranes with TBS-T containing 5% nonfat dry milk and further incubation with anti-ACTB or anti-GAPDH antibodies (Supplementary Table S2).

### Immunohistochemistry

FA transporter proteins were localized at the placenta-endometrium interface using a procedure described previously^29^. Briefly, 5 μm sections were mounted on SuperFrost Plus microscope slides (Menzel-Gläzer; Braunschweig, Germany), heated and washed in xylene. After rehydration, the antigen retrieval was performed by heating slides in citrate buffer (10 mM sodium citrate, 0.05% Tween 20; pH 6.0) for 15 min. Afterwards, 30% hydrogen peroxidase in methanol was added for 15 min followed by 1 h treatment of slides with a Fish Serum Blocking Buffer (37527; ThermoFisher Scientific). Then, an overnight incubation at 4 °C with the following antibodies was performed: rabbit anti-SLC27A1 (A12847; ABclonal Germany GmbH, Dusseldorf, Germany; 1:200), rabbit anti-SLC27A4 (A16101; ABclonal; 1:100), or rabbit anti-SLC27A6 (SAB102195; Sigma-Aldrich; 1:50). The next day, sections were incubated for 30 min with a goat anti-rabbit IgG secondary antibody (BA-1000; Vector Laboratories, Inc., Burlingame, CA, USA; 1:2000), washed with TBS, and treated with a mixture of Reagents A and B from VECTASTAIN® Elite® ABC_HRP kit, Peroxidase Rabbit IgG (PK-6101; Vector Laboratories). Subsequently, slides were treated with 3,3′-diamidinobenzidine (D5637; Sigma-Aldrich), counterstained with hematoxylin, dehydrated, and mounted using DPX (06522; Sigma-Aldrich). Negative controls were carried out by replacing primary antibodies with a rabbit IgG negative control (I-1000; Vector Laboratories) according to the manufacturer’s instructions. Slides were photographed using Zeiss AXIO Imager.Z1 microscope (Carl Zeiss Microscopy GmbH, Jena, Germany).

### Statistical analyses

All statistical analyses were conducted using GraphPad Prism v. 10.4.1. (GraphPad Software, Inc., San Diego, CA, USA). The Shapiro–Wilk test was applied to assess the normal distribution of the data. The data that did not pass the normality test were log-transformed and analyzed using non-parametric tests: Kruskal–Wallis test for unpaired data or Friedman test for paired data. The data that passed the normality test were further examined for the homogeneity of variance using Bartlett’s test. Depending on the results of this test, the data were further analyzed using one-way ANOVA or Welch ANOVA for multiple groups. Data from two groups were analyzed using Student’s *t*-test. All numerical data are presented as means ± SEM, and means were considered to be statistically different at *p* ≤ 0.05 with a tendency estimated at 0.05 < *p* ≤ 0.08.

## Supplementary Information

Below is the link to the electronic supplementary material.

Supplementary Material 1

## Data Availability

The datasets generated and analyzed during the current study are available in the Repository for Open Data (RepOD) 10.18150/E7BXCB.
