# Supplementary material for: Fatty acid transporters in the porcine conceptus and early placenta and the effects of polyunsaturated fatty acids on trophoblast cells
Source: Sci Rep. 2026 May 2;16:20376. doi: 10.1038/s41598-026-51024-w (PMC13328565; doi:10.1038/s41598-026-51024-w)
Supplement: Supplementary file 1 — Supplementary Material 1 [file 41598_2026_51024_MOESM1_ESM.pdf]

Fatty acid transporters in the porcine conceptus and early placenta and the effects of polyunsaturated fatty acids on trophoblast cells

Agnieszka Blitek* and Magdalena Szymanska

InLife Institute of Animal Reproduction and Food Research, Polish Academy of Sciences, Trylinskiego 18, 10-683 Olsztyn, Poland

*Corresponding author: a.blitek@pan.olsztyn.pl

Supplementary Table S1. Abbreviations and full names of genes, and the ID numbers of TaqMan probes applied to examine the relative mRNA expression using Real-time PCR.

Abbreviation	Gene name	ID of TaqMan probe
ACOX1	acyl-CoA oxidase 1	Ss03386405_u1
ANGPT1	angiopoietin 1	Ss03391079_m1
ANGPT2	angiopoietin 2	Ss03392362_m1
ANGPTL4	angiopoietin like 4	Ss03379927_u1
CD36	CD36 molecule; fatty acid translocase	Ss03388549_m1
CPT1A	carnitine palmitoyltransferase 1A	Ss03373366_m1
CYP11A1	cytochrome P450 family 11 subfamily A member 1	Ss03384849_u1
FABP3	fatty acid binding protein 3	Ss03386206_g1
FABP5	fatty acid binding protein 5	Ss03392151_m1
LDLR	low density lipoprotein receptor	Ss03374441_u1
PPARA	peroxisome proliferator activated receptor alpha	Ss03380164_u1
PPARD	peroxisome proliferator activated receptor delta	Ss03394198_g1
PPARG	peroxisome proliferator activated receptor gamma	Ss03394829_m1
PTGES	prostaglandin E synthase	Ss03392129_m1
PTGIS	prostaglandin I2 synthase	Ss03374149_m1
PTGS2	prostaglandin-endoperoxide synthase 2	Ss03394695_g1
SLC27A1	solute carrier family 27 member 1	Ss03388764_m1
SLC27A2	solute carrier family 27 member 2	Ss03373885_m1
SLC27A3	solute carrier family 27 member 3	Ss06872017_g1
SLC27A4	solute carrier family 27 member 4	Ss04329561_m1
SLC27A6	solute carrier family 27 member 6	Ss06904004_m1
STAR	steroidogenic acute regulatory protein	Ss03381250_u1
VEGFA	vascular endothelial growth factor A	Ss03393993_m1
ACTG1	actin gamma 1; reference gene	Ss03376081_u1
HPRT1	hypoxanthine phosphoribosyltransferase 1; reference gene	Ss03388274_m1
GAPDH	glyceraldehyde-3-phosphate dehydrogenase; reference gene	Ss03375435_u1

Supplementary Table S2. Information on primary antibodies applied in Western blot analysis.

Peptide/Protein	Name of antibody	Catalog no. / manufacturer	Species raised in	Dilution used
ANGPT1	Anti-Angiopoietin 1 antibody	ab95230 / abcam	rabbit	1:500
ANGPT2	Anti-Angiopoietin 2/ANG2 antibody	ab64015 / abcam	rabbit	1:500
CD36	CD36/SR-B3 Rabbit pAb	A5792 / ABclonal	rabbit	1:500
PPARG	PPARγ Antibody (H100)	Sc-7196 / Santa Cruz Biotechnology	rabbit	1:100
PTGES	Prostaglandin E Synthase-1 (microsomal) Polyclonal Antibody	160140 / Cayman Chemical	rabbit	1:200
PTGIS	Prostaglandin I Synthase Polyclonal Antibody	160640 / Cayman Chemical	rabbit	1:200
PTGS2	COX2/PTGS2 Rabbit pAb	A1253 / ABclonal	rabbit	1:500
SLC27A1	SLC27A1 Rabbit pAb	A12847 / ABclonal	rabbit	1:1,000
SLC27A4	Anti-SLC27A4/FATP4 antibody [EPR17319] – C terminal	ab199719 / abcam	rabbit	1:500
SLC27A6	SLC27A6 Rabbit pAb	A24512 / ABclonal	rabbit	1:500
ACTB	Anti-beta Actin antibody	ab8227 / abcam	rabbit	1:2,000
GAPDH	GAPDH Rabbit mAb (High Dilution)	A19056 / ABclonal	rabbit	1:10,000

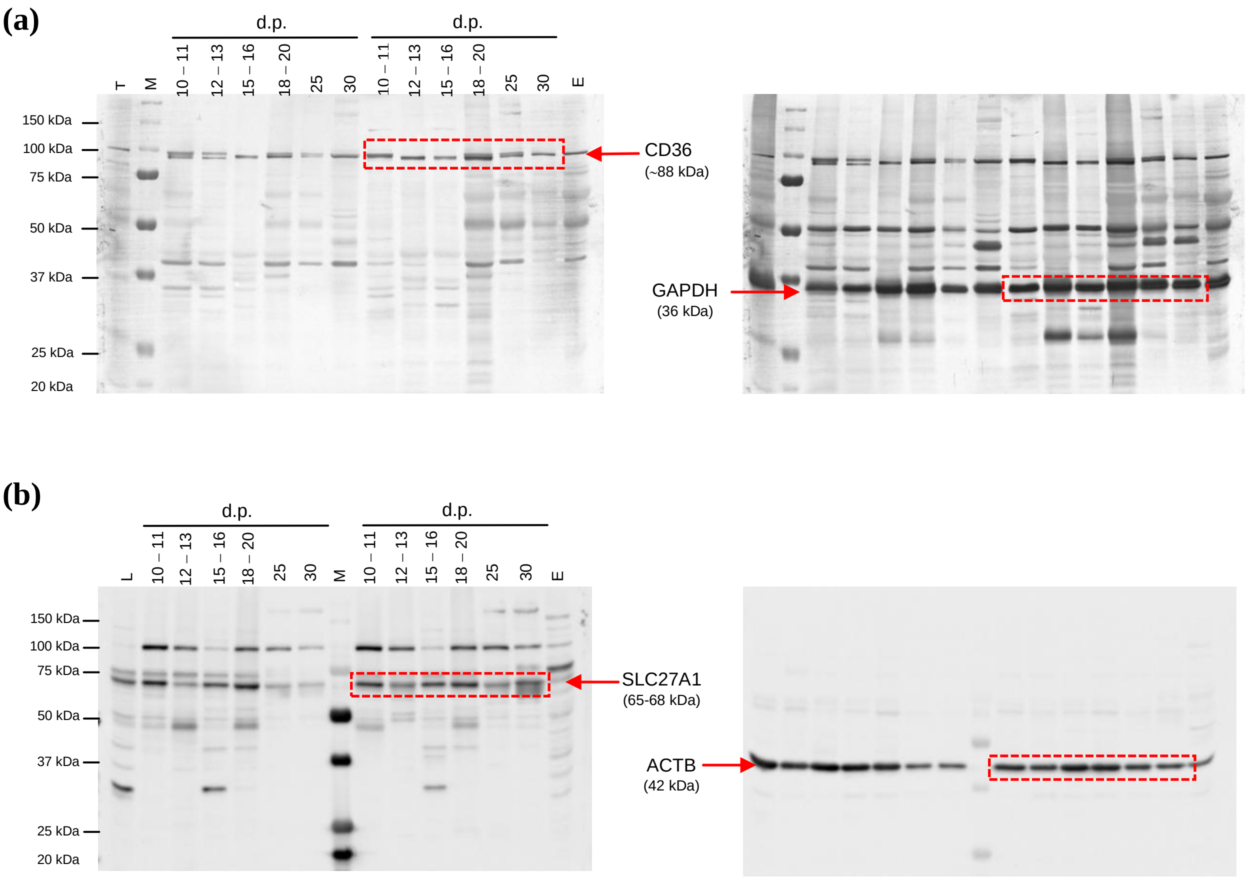

Supplementary Fig. S1. Western blot results presenting CD36 (**a**) and SLC27A1 (**b**) protein expression in conceptuses and placentae collected on days 10-11, 12-13, 15-16, 18-20, 25, and 30 of pregnancy (left panel). Glyceraldehyde-3-phosphate dehydrogenase (GAPDH) and β-actin (ACTB) were used as an internal controls of protein loading for CD36 and SLC27A1, respectively. After being photographed, blots presented in the left panel were re-probed for GAPDH or ACTB, and photographed again (right panel). Testis (T), liver (L), and endometrium (E) were used as positive tissue controls for CD36 and/or SLC27A1 proteins. M, marker; d.p., days of pregnancy. The red dashed lines show fragments of each blot presented in Fig. 2.

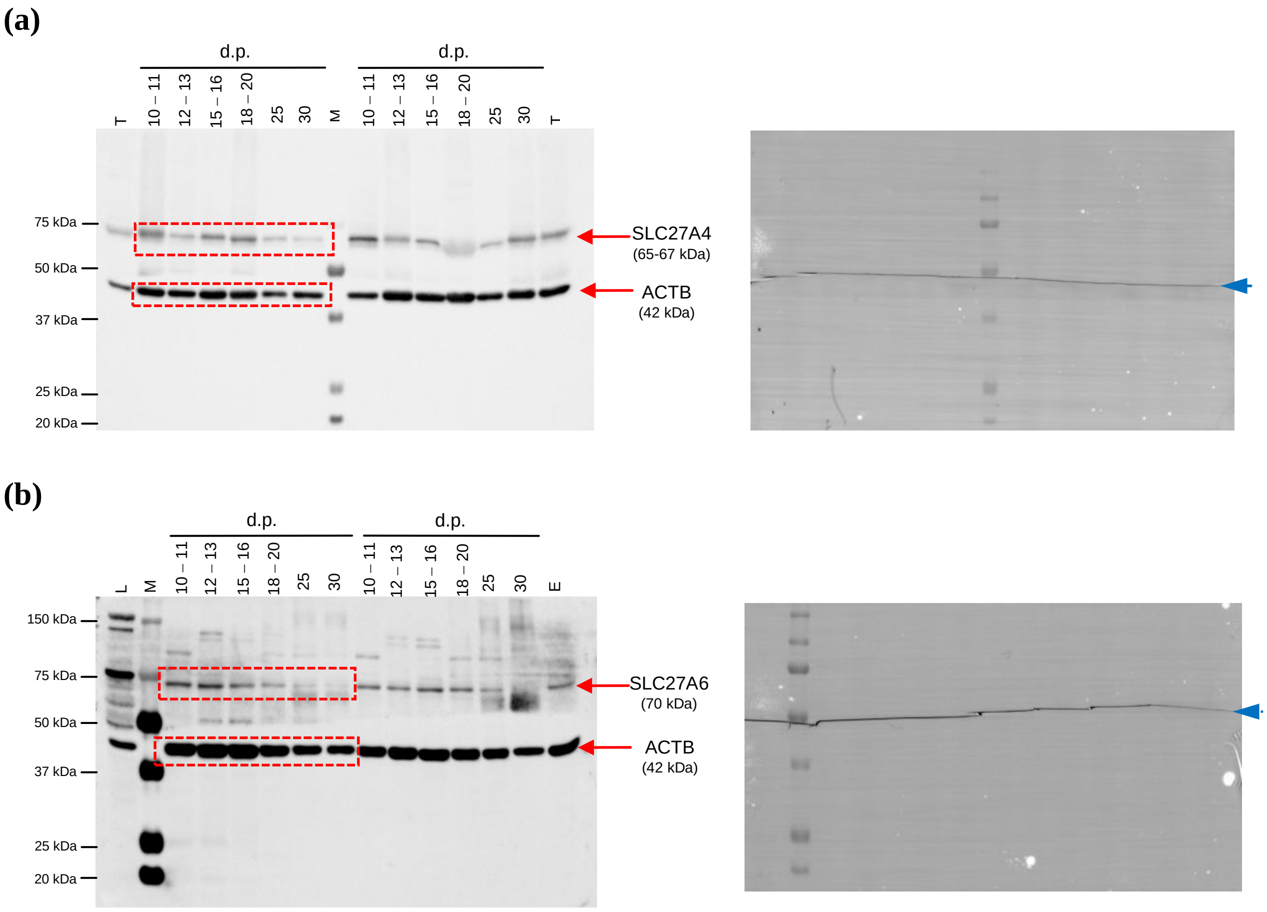

Supplementary Fig. S2. Western blot results presenting SLC27A4 (**a**) and SLC27A6 (**b**) protein expression in conceptuses and placentae collected on days 10-11, 12-13, 15-16, 18-20, 25, and 30 of pregnancy (left panel). β-actin (ACTB) was used as an internal control of protein loading. Membranes were cut after transfer at about 50 kDa (marked in the right panel with blue arrows) and the upper part was incubated with anti-SCL27A4 or anti-SLC27A6 antibodies, whereas the lower part was incubated with an anti-ACTB antibody. Testis (T), liver, (L), and endometrium (E) were used as positive tissue controls for SLC27A proteins. M, marker; d.p., days of pregnancy. The red dashed lines show the fragment of each blot presented in Fig. 2.

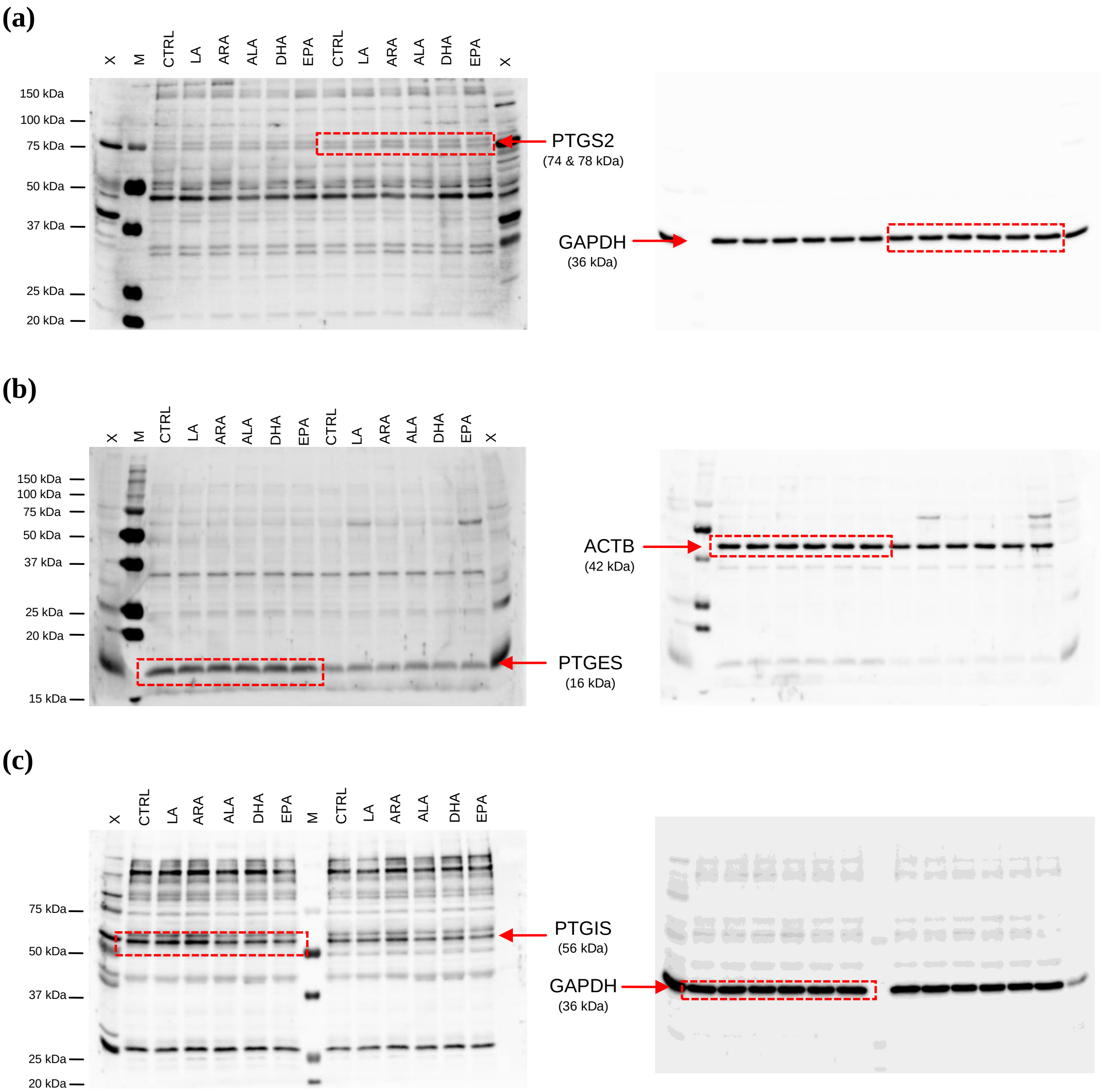

Supplementary Fig. S3. Western blot results presenting prostaglandin-endoperoxide synthase (PTGS2; **a**), prostaglandin E synthase (PTGES; **b**), and prostaglandin I2 synthase (PTGIS; **c**) protein expression in porcine trophoblast cells exposed to linoleic acid (LA), arachidonic acid (ARA), α-linolenic acid (ALA), docosahexaenoic acid (DHA), or eicosapentaenoic acid (EPA; left panel). β-actin (ACTB) or glyceraldehyde-3-phosphate dehydrogenase (GAPDH; right panel) were used as an internal control of protein loading. After being photographed, blots presented in the left panel were re-probed for ACTB or GAPDH, and photographed again. M, marker; CTRL, control cells not exposed to PUFAs; X, unrelated samples (randomly selected samples applied in marginal paths to avoid excessive bending of bands at the edge of gels). The red dashed lines show the fragment of each blot presented in Fig. 4.

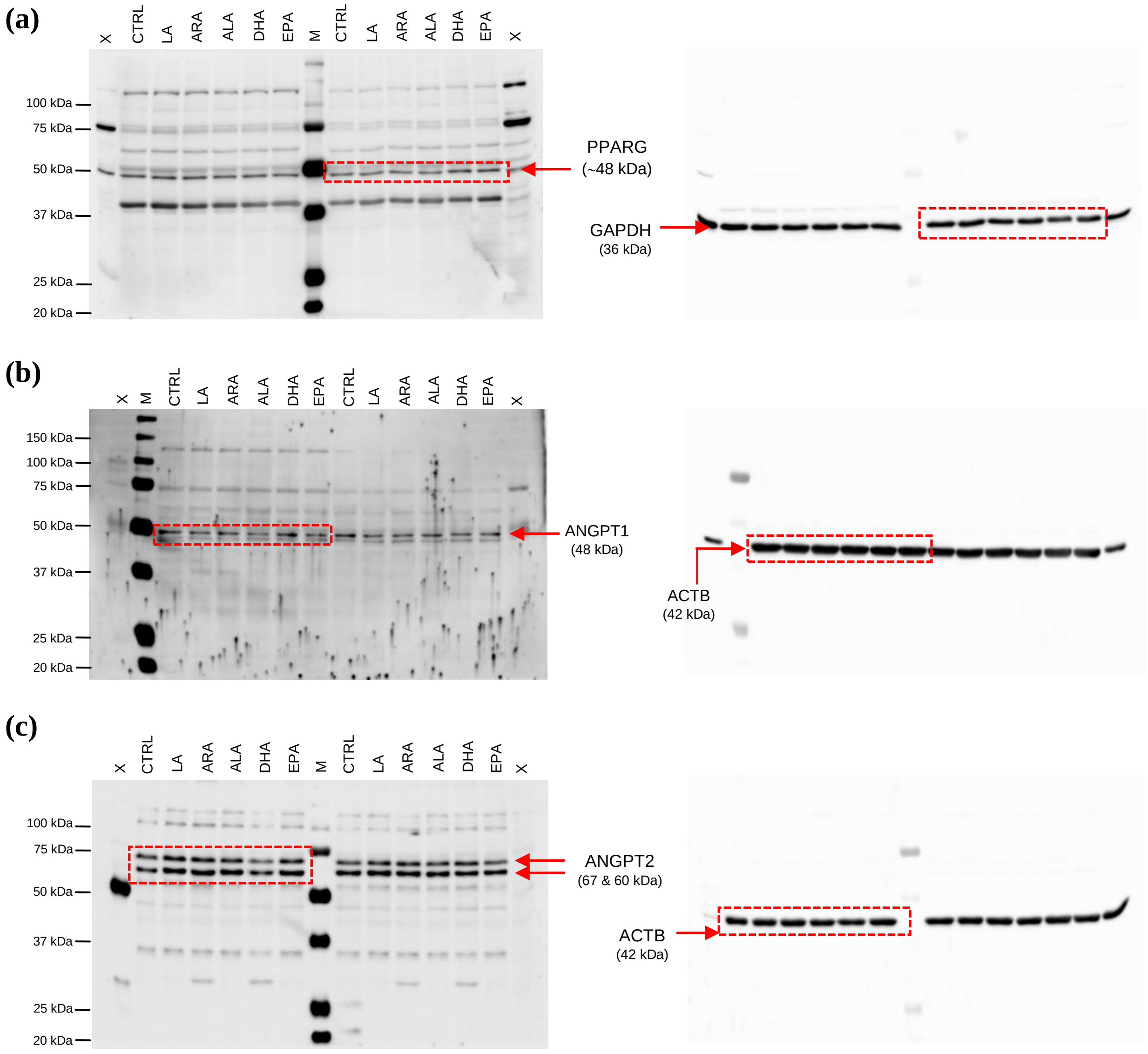

Supplementary Fig. S4. Western blot results presenting peroxisome proliferator-activated receptor gamma (PPARG; **a**), angiopoietin 1 (ANGPT1; **b**), and 2 (ANGPT2; **c**) protein expression in porcine trophoblast cells exposed to linoleic acid (LA), arachidonic acid (ARA), α-linolenic acid (ALA), docosahexaenoic acid (DHA), or eicosapentaenoic acid (EPA; left panel). β-actin (ACTB) or glyceraldehyde-3-phosphate dehydrogenase (GAPDH; right panel) were used as an internal control of protein loading. After being photographed, blots presented in the left panel were re-probed for ACTB or GAPDH, and photographed again. M, marker; CTRL, control cells not exposed to PUFAs; X, unrelated samples (randomly selected samples applied in marginal paths to avoid excessive bending of bands at the edge of gels). The red dashed lines show the fragment of each blot presented in Fig. 4.
